# High correlation between *in vivo* [^123^I]β-CIT SPECT/CT imaging and post-mortem immunohistochemical findings in the evaluation of lesions induced by 6-OHDA in rats

**DOI:** 10.1186/2191-219X-3-46

**Published:** 2013-06-10

**Authors:** Susanne Bäck, Mari Raki, Raimo K Tuominen, Atso Raasmaja, Kim Bergström, Pekka T Männistö

**Affiliations:** 1 Division of Pharmacology and Toxicology, Faculty of Pharmacy, University of Helsinki, PO Box 56 (Viikinkaari 5E), Helsinki FI-00014, Finland; 2 Centre for Drug Research, Faculty of Pharmacy, University of Helsinki, PO Box 56 (Viikinkaari 5E), Helsinki FI-00014, Finland

**Keywords:** 6-OHDA, β-CIT, Rat, SPECT

## Abstract

**Background:**

6-Hydroxydopamine (6-OHDA) is widely used in pre-clinical animal studies to induce degeneration of midbrain dopamine neurons to create animal models of Parkinson's disease. The aim of our study was to evaluate the potential of combined single-photon emission computed tomography/computed tomography (SPECT/CT) for the detection of differences in 6-OHDA-induced partial lesions in a dose- and time-dependent manner using the dopamine transporter (DAT) ligand 2β-carbomethoxy-3β-(4-[^123^I]iodophenyl)tropane ([^123^I]β-CIT).

**Methods:**

Rats were unilaterally lesioned with intrastriatal injections of 8 or 2 × 10 μg 6-OHDA. At 2 or 4 weeks post-lesion, 40 to 50 MBq [^123^I]β-CIT was administered intravenously and rats were imaged with small-animal SPECT/CT under isoflurane anesthesia. The striatum was delineated and mean striatal activity in the lesioned side was compared to the intact side. After the [^123^I]β-CIT SPECT/CT scan, the rats were tested for amphetamine-induced rotation asymmetry, and their brains were immunohistochemically stained for DAT and tyrosine hydroxylase (TH). The fiber density of DAT- and TH-stained striata was estimated, and TH-immunoreactive cells in the rat substantia nigra pars compacta (SNpc) were stereologically counted.

**Results:**

The striatal uptake of [^123^I]β-CIT differed significantly between the lesion groups and the results were highly correlated to both striatal DAT- and TH-immunoreactive fiber densities and to TH-immunoreactive cell numbers in the rat SNpc. No clear progression of the lesion could be seen.

**Conclusions:**

[^123^I]β-CIT SPECT/CT is a valuable tool in predicting the condition of the rat midbrain dopaminergic pathway in the unilateral partial 6-OHDA lesion model of Parkinson's disease and it offers many advantages, allowing repeated non-invasive analysis of living animals.

## Background

The main feature of Parkinson's disease (PD) is degeneration of the nigrostriatal dopaminergic neurons, resulting in motor symptoms such as resting tremor, posture instability, rigidity, and bradykinesia [1]. The neurotoxin 6-hydroxydopamine (6-OHDA) is widely used in a well-established rat model of PD to mimic the midbrain dopaminergic degeneration seen in the disease [2-5]. The extent of the lesion is dependent on the 6-OHDA injection site as well as on the dose of 6-OHDA [6,7]. When injected intrastriatally, 6-OHDA causes immediate damage to the dopaminergic fibers in the striatum followed by a retrograde progressive loss of dopaminergic cell bodies in the substantia nigra pars compacta (SNpc) [8]. This rat partial lesion model of PD is widely used in pre-clinical studies since the progressive dying back of the dopamine neurons provides a time window that allows for testing of potentially neuroprotective agents.

It would be important to follow the degree of lesion and efficacy of treatments in living animals as a function of time. Conventionally, this is done by monitoring motor functions with behavioral tests or by invasive methods, such as *in vivo* microdialysis. To do more extensive evaluation of the dopaminergic system, endpoint tissue analyses usually need to be applied. *In vivo* imaging of 6-OHDA-lesioned rats with single-photon emission computed tomography/computed tomography (SPECT/CT) offers a considerable potential for monitoring changes in the midbrain dopaminergic pathway allowing longitudinal studies in living animals.

In this study, we used the high-affinity dopamine transporter (DAT) radioligand 2β-carbomethoxy-3β-(4-[^123^I]iodophenyl)tropane ([^123^I]β-CIT) [9,10] to estimate the DAT density in the rat striatum in unilaterally 6-OHDA-lesioned rats. DAT is responsible for the termination of dopamine signaling by re-uptake of dopamine from the synaptic cleft [11]. In the CNS, the transporter is found only in the plasma membrane of dopamine neurons [12] which makes it an excellent marker of this network.

To our knowledge, only a few studies examining DAT binding with SPECT in 6-OHDA-lesioned rats have been published [13-15]. In these studies, 6-OHDA was administered to the rat SN [13,15] or medial forebrain bundle (MFB) [14]. Compared to intrastriatal injections of 6-OHDA, injections to the SN or MFB result in a more extensive dopaminergic lesion that is almost fully developed in less than 1 week after the injection [4,5,7]. Several studies of the rat unilateral 6-OHDA lesion model have been conducted using positron emission tomography (PET) cameras and tracers [16-23], but these studies have also mainly focused on the SN or MFB 6-OHDA lesion. Furthermore, there are very few studies presenting data on the degree of correlation between *in vivo* imaging of DAT binding and immunohistochemical findings. Therefore, the aim of our study was to determine the discrimination capacity of [^123^I]β-CIT SPECT/CT in terms of severity of dopaminergic lesion and time after induction of lesion following intrastriatal administration of 6-OHDA. We also wanted to assess the degree of correlation between [^123^I]β-CIT SPECT/CT and immunohistochemical data used for the evaluation of the rat midbrain dopaminergic system in the unilateral partial 6-OHDA lesion model of PD.

## Methods

### Animals and surgery

Wistar male rats (Harlan, The Netherlands) were group-housed in a 12:12 h light/dark cycle. The rats had free access to rodent food (Harlan) and tap water. All procedures were approved by the National Animal Experiment Board (ESAVI/4706/04.10.03/2011) and carried out in accordance with the European Communities Council Directive 86/609/EEC.

Nineteen rats (250 to 300 g) received unilateral intrastriatal injections of 6-OHDA (6-OHDA hydrochloride, Sigma-Aldrich, St. Louis, MO, USA) in a stereotaxic operation. The rats were anesthetized with isoflurane (2% to 4%) and placed in a stereotaxic frame (Stoelting, Wood Dale, IL, USA). After exposure of the skull, the coordinates for single-site (1.0 mm anterior and 2.7 mm lateral to bregma) and two-site injections (1.6 mm anterior, 2.2 mm lateral, and 0.4 mm posterior, 4.0 mm lateral to bregma) were determined according to the rat brain atlas of Paxinos and Watson [24]. Injections of 8 μg (single-site) or 2 × 10 μg (two-site) 6-OHDA diluted in 0.02% ascorbic acid were made 5 mm below the dura using a stereotaxic injector (Stoelting, Wood Dale, IL, USA) and a 10-μl syringe (Hamilton, Bonaduz, Switzerland). All injections were done with an injection volume of 4 μl and injection speed of 1 μl/min. After the injection, the needle was kept in place 2 min before withdrawal to prevent reflux. During the operation, all rats received an injection of tramadol (1 mg/kg, s.c., Tramal, Orion Oyj, Espoo, Finland) for post-operative pain, and the rats were single-housed overnight. Four additional rats were left intact and used for assessment of basal values. Intact animals were used since in a previous small pilot study we did not detect any change in [^123^I]β-CIT binding following sham lesion (results not shown).

### SPECT/CT imaging

At 2 or 4 weeks post-lesion, the rats (290 to 350 g) received an intravenous injection of 40 to 50 MBq [^123^I]β-CIT (MAP Medical Technologies Oy, Tikkakoski, Finland). Four hours later, the rats were imaged with nanoSPECT/CT (Bioscan Inc., Washington, DC, USA) featuring 2.5-mm multipinhole rat apertures. Imaging was performed under isoflurane anesthesia (2% to 3%), and the body temperature was maintained warm using a heated animal bed (Minerve, France). Brain SPECT images were collected in 24 projections using time per projection of 150 s resulting in a total acquisition time of 30 min. CT imaging was carried out with a 45-kVp tube voltage in 180 projections. SPECT images were reconstructed with HiSPECT NG software (Scivis GmbH, Germany) and fused with CT datasets using InVivoScope software (Bioscan Inc.). Reconstructed SPECT images were reoriented and analyzed with InVivoScope software using CT data as a reference. Elliptical volume of interest was defined around the striatum of an intact rat and used in all subsequent analyses. The mean striatal activity (activity-to-volume ratio) was counted and corrected for background activity from the cerebellum. Two of the intact rats were imaged at each time point, and the results from the four intact animals were pooled together in all subsequent analyses.

### Amphetamine-induced rotations

Two days after each SPECT/CT scan, rats were injected with d-amphetamine (2.5 mg/kg, intraperitoneally (i.p.)), and full amphetamine-induced turns were monitored for 120 min using Rotorat software (Med Associates Inc., St. Albans, VT, USA). The results were counted as the total amount of net ipsilateral rotations per 120 min.

### Tissue preparation and immunohistochemistry

One day after the drug-induced rotational test, rats were anesthetized with an overdose of pentobarbital (90 mg/kg, i.p., Mebunat, Orion Oyj, Espoo, Finland). Rat brains were fixed by intracardial perfusion with 4% paraformaldehyde for 10 min. The brains were then removed and post-fixed in 4% paraformaldehyde overnight. The rat brains were stored in 20% sucrose at 4°C until freezing, and frozen brains were cut on a microtome into 40-μm sections in series of six.

Fixed free-floating sections were washed with PBS and treated with 3% hydrogen peroxide solution. For anti-DAT staining, the sections were incubated in 10 mM citrate buffer (pH 6) at 80°C for 30 min. After incubation in the blocking solution (2% normal horse serum or normal rabbit serum, respectively, Vector Labs, Burlingame, CA, USA), the sections were incubated overnight in mouse anti-tyrosine hydroxylase (TH) antibody (1:2000, #MAB318, Millipore, Temecula, CA, USA) or rat anti-DAT antibody (1:1000, #MAB369, Millipore). Following incubation in biotinylated secondary antibody (1:200, horse anti-mouse #BA2001 or rabbit anti-rat #BA4000, Vector labs), the staining was reinforced with avidin-biotin complex (ABC-kit, Vector Labs) and visualized with 3′3-diaminobenzidine (Sigma-Aldrich).

### Stereologic assessment of TH-reactive cells in the SNpc

The number of TH-reactive cells in the SNpc was estimated with stereology using the Stereo Investigator platform (MicroBrightField, Williston, VT, USA). Six nigral sections (every sixth section) from each rat brain were chosen (ranging from approximately 4.5 to 6.0 mm posterior to bregma[24]) and the cells were counted bilaterally in a blinded fashion using unbiased counting rules.

### Estimation of optical density of dopaminergic fibers in the rat striatum

Optical density of TH- and DAT-stained striata was measured using ImagePro Plus software (Media Cybernetics, Bethesda, MD, USA). Five pictures from the rat striatum (ranging from approximately 1.6 to 0.2 mm anterior to bregma, [24]) were taken with a digital camera (Nikon Corporation, Tokyo, Japan) attached to a stereomicroscope. The dorsal striatum was delineated with a circle tool, and the optical density inside the area of interest was measured. The same area of interest was used for all sections, and all values were corrected by subtracting the background value achieved from the cortex of each section.

### Statistics

All results are given as mean ± standard error of the mean (SEM). All data were tested for normal distribution with Levene's test for homogenicity. In the case of multiple treatment groups, the data were analyzed using one-way analysis of variance (ANOVA) followed by Tukey *post hoc* test. The effect of the 6-OHDA-induced lesion as compared to the corresponding intact side was tested with paired samples *t* test. Correlations between measurements were tested with Pearson correlation test. All statistical analyses were done with Pasw Statistics 18 software (SPSS, Inc., Chicago, IL, USA). A *P* value <0.05 was considered to be statistically significant.

## Results

### DAT binding of [^123^I]β-CIT measured with nanoSPECT/CT

**Figure 1:**
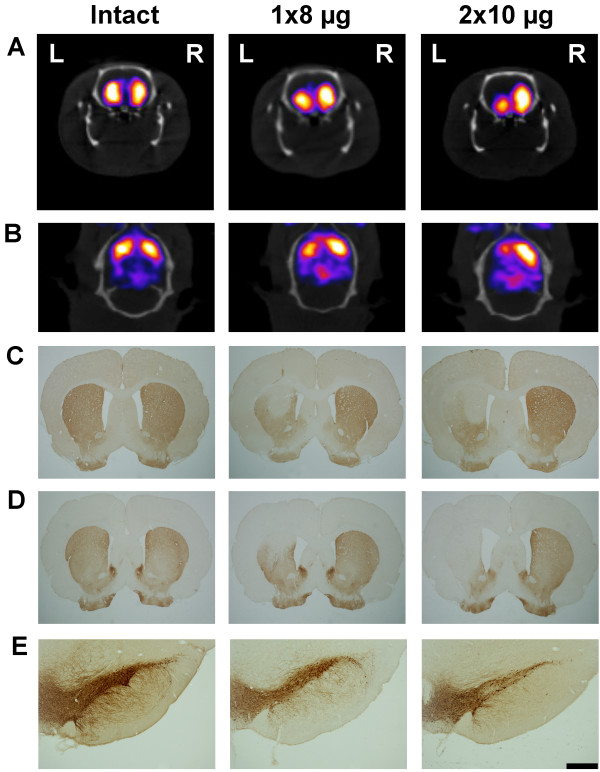
**Representative pictures of striatal binding of [**^**123**^**I]β-CIT and immunohistochemical staining with dopaminergic markers.** Striatal activity of [^123^I]β-CIT detected with SPECT/CT (coronal view (**A)** and transversal view (**B**)), DAT immunoreactivity in the striatum (**C**), TH immunoreactivity in the striatum (**D**), and nigral TH-immunoreactive cells (**E**) in intact and 6-OHDA-lesioned rats (8 and 2 × 10 μg 6-OHDA) at 4 weeks post-lesion. The orientation of the pictures is denoted in (**A**) with left (L) and right (R). Scale bar in (**E**) is 500 μm.

Unilateral intrastriatal injection of 6-OHDA resulted in a statistically significant decrease in the striatal [^123^I]β-CIT activity (MBq/mm^3^) in the lesioned (left) side as compared to the corresponding intact (right) side in all lesion groups (paired samples *t* test) (Table 1; representative pictures in Figure 1A,B). No difference in the right side [^123^I]β-CIT binding was detected between the treatment groups (one-way ANOVA (*P* = 0.655, *F*_4,21_ = 0.618)).

**Table 1:** **Quantitative results from [**^**123**^**I]β-CIT SPECT/CT and TH-reactive cell counts**

		**Intact**		**8 μg 6-OHDA**		**2 × 10 μg 6-OHDA**	
		**Left**	**Right**	**Left**	**Right**	**Left**	**Right**
Mean striatal uptake of [^123^I]β-CIT (×10^-4^ MBq/mm^3^)	2 weeks	2.3 ± 0.3	2.1 ± 0.3	2.0 ± 0.2*	2.6 ± 0.3	1.4 ± 0.2**	2.2 ± 0.2
	4 weeks			1.7 ± 0.1**	2.2 ± 0.1	1.4 ± 0.1**	2.3 ± 0.2
TH-reactive cells in the SNpc	2 weeks	8,047 ± 1,074	8,675 ± 487	5,426 ± 586*	8,232 ± 164	2,894 ± 312**	8,328 ± 439
	4 weeks			5,759 ± 640*	8,298 ± 338	2,658 ± 655**	8,239 ± 434

Striatal [^123^I]β-CIT binding detected with SPECT/CT and TH-reactive cell numbers in the SNpc at 2 and 4 weeks after induction of 6-OHDA-mediated degeneration of the rat midbrain dopaminergic pathway. Unilateral injection of 6-OHDA (8 or 2 × 10 μg) to the left rat striatum resulted in a significant decrease in striatal [^123^I]β-CIT uptake and nigral TH-reactive cell counts when compared to the right (intact) side. The results are given as mean ± SEM. *P < 0.05 and **P < 0.01 as compared to the right (intact) side (paired samples *t* test, *n* = 4 to 5 per group).

**Figure 2:**
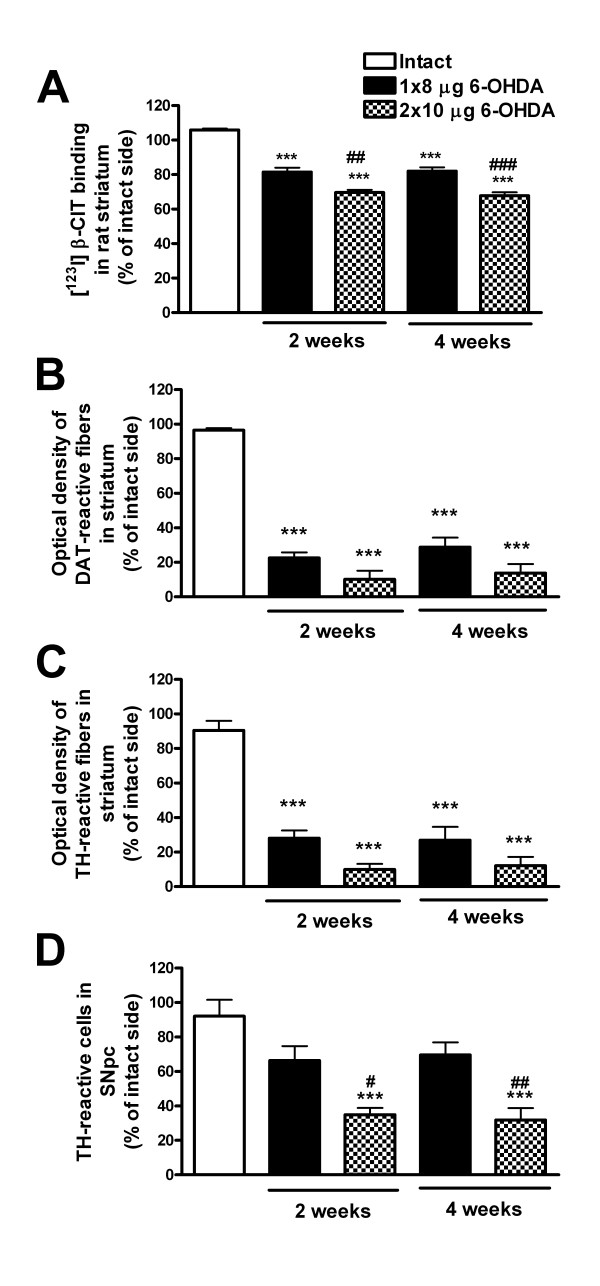
**Unilateral 6-OHDA-induced changes in [**^**123**^**I]β-CIT SPECT/CT and immunohistochemical findings.** All results are presented as percentage of the lesioned/left side versus the intact/right side. (**A**) The 6-OHDA injections caused a significant decrease in striatal [^123^I]β-CIT uptake as compared to intact rats at both 2 and 4 weeks post-lesion. Furthermore, the two different lesions (8 and 2 × 10 μg) could be distinguished with [^123^I]β-CIT SPECT/CT. Intrastriatal injection of 8 or 2 × 10 μg 6-OHDA resulted in extensive loss of (**B**) DAT- and (**C**) TH-immunoreactive fibers in the striatum, but there was no significant difference between the two lesion groups. However, when counting (**D**) TH-reactive cells in the substantia nigra pars compacta, the cell loss significantly differed between the lesion types. All results are shown as mean ± SEM. ***P < 0.001 as compared to intact rats; ^#^*P* < 0.05, ^##^*P* < 0.01, and ^###^*P* < 0.001 as compared to 8-μg 6-OHDA lesion group (one-way ANOVA followed by Tukey *post hoc* test, *n* = 4 to 5 per group).

The 6-OHDA-induced changes in the striatal [^123^I]β-CIT uptake (percentage of left versus right side) showed statistically significant differences between all groups (intact, 8 μg 6-OHDA, and 2 × 10 μg 6-OHDA) at both 2 and 4 weeks post-lesion (one-way ANOVA (*P* < 0.001, *F*_4,21_ = 57.460) and Tukey *post hoc* test) (Figure 2A). There was a decrease in striatal [^123^I]β-CIT activity in the 6-OHDA-lesioned hemisphere of 18% ± 2% (at both 2 and 4 weeks) in the 8-μg 6-OHDA lesion group and 30% ± 2% (at 2 weeks) and 32% ± 2% (at 4 weeks) in the 2 × 10-μg 6-OHDA group. Thus, the decrease in DAT binding due to the 6-OHDA-induced lesion was of the same magnitude at both 2 and 4 weeks post-lesion.

### Amphetamine-induced rotation asymmetry

**Figure 3:**
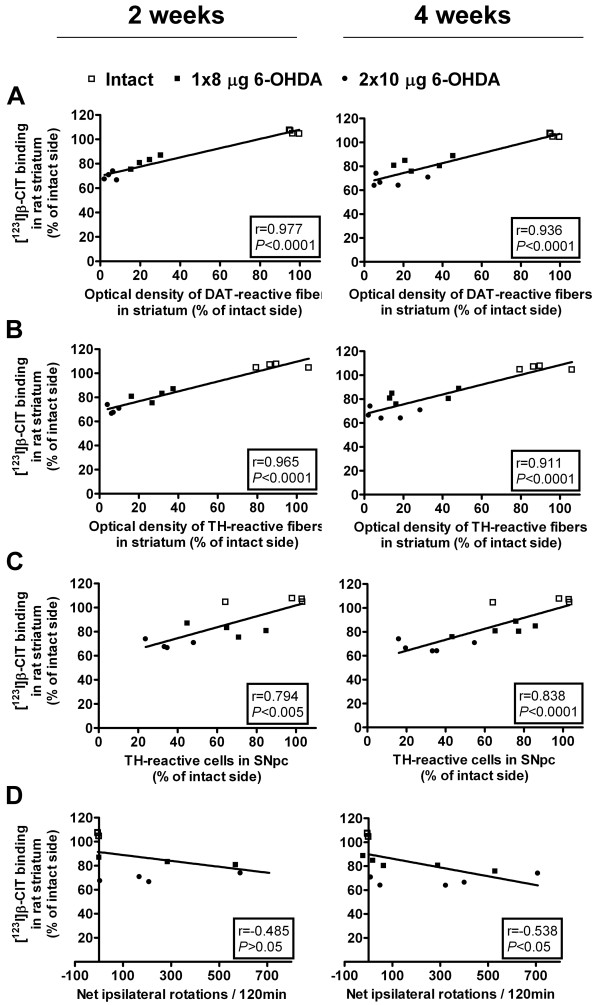
**Correlations between [**^**123**^**I]β-CIT SPECT/CT and immunohistochemical and behavioral data at 2 and 4 weeks post-lesion.** Striatal binding of [^123^I]β-CIT showed high correlation to striatal (**A**) DAT- and (**B**) TH-reactive fiber densities and (**C**) nigral TH-reactive cell numbers at both time points. At 4 weeks, [^123^I]β-CIT SPECT/CT correlated moderately to the amount of (**D**) amphetamine-induced rotations, whereas no significant correlation was found at 2 weeks post-lesion. Correlation coefficients (*r*) and statistical significance (*P*) were determined with Pearson correlation test (*n* = 12 (2 weeks) and *n* = 14 (4 weeks)).

Following an i.p. injection of amphetamine, no statistically significant differences in the drug-induced rotational behavior could be detected between the treatment groups (one-way ANOVA, *P* = 0.211, *F*_4,22_ = 1.628). At 2 weeks post-lesion, the amount of net ipsilateral rotations per 120 min was 456 ± 207 in the 8-μg lesion group and 242 ± 119 for rats lesioned with 2 × 10 μg 6-OHDA (*n* = 4 per group). At 4 weeks post-lesion, the corresponding results were 175 ± 104 and 298 ± 127, respectively (*n* = 5 per group). For correlations with other results, see Table 2 and Figure 3. The intact rats showed no turning preference (-3 ± 1 net ipsilateral turns per 120 min, *n* = 4).

**Table 2:** Correlations between different measurements

		**SPECT**	**Rotations**	**TH cells**	**TH fibers**	**DAT fibers**
2 weeks	SPECT	*1*	*-0.485*	*0.794*	*0.965*	*0.977*
			*P* > 0.05	*P* < 0.005	*P* < 0.0001	*P* < 0.0001
	Rotations	-	*1*	*0.089*	*0.462*	*0.499*
				*P* > 0.05	*P* > 0.05	*P* > 0.05
	TH cells	-	-	*1*	*0.724*	*0.752*
					*P* < 0.01	*P* < 0.005
	TH fibers	-	-	-	*1*	*0.981*
						*P* < 0.0001
	DAT fibers	-	-	-	-	*1*
4 weeks	SPECT	*1*	*-0.538*	*0.838*	*0.911*	*0.936*
			*P* < 0.05	*P* < 0.0001	*P* < 0.0001	*P* < 0.0001
	Rotations	-	*1*	*0.754*	*0.656*	*0.630*
				*P* < 0.005	*P* < 0.05	*P* < 0.05
	TH cells	-	-	*1*	*0.731*	*0.769*
					*P* < 0.005	*P* < 0.005
	TH fibers	-	-	-	*1*	*0.985*
						*P* < 0.0001
	DAT fibers	-	-	-	-	*1*

Correlation coefficients (italic numbers) and statistical significance (*P*) were determined with Pearson correlation (*n* = 12 to 13 at 2 weeks post-lesion, and *n* = 14 at 4 weeks post-lesion).

### Morphometric analysis of TH-reactive cell bodies in SNpc and striatal fiber density

Intrastriatal injections of 8 and 2 × 10 μg 6-OHDA resulted in statistically significant loss of TH-reactive cells in the left side of the rat SNpc as compared to the right side (paired samples *t* test) (Table 1; representative pictures in Figure 1E). There was no difference in the right side TH-reactive cell numbers between the different groups (one-way ANOVA (*P* = 0.655, *F*_4,21_ = 0.618)). The remaining number of TH-reactive cells following injection of 2 × 10 μg 6-OHDA (35% ± 4% at 2 weeks and 32% ± 7% at 4 weeks) was significantly lower as compared to intact rats (92% ± 9%) and as compared to the 8-μg lesion (66% ± 8% at 2 weeks and 70% ± 7% at 4 weeks) at both 2 and 4 weeks post-lesion (TH-reactive cell number in the left side as compared to the right side, one-way ANOVA (*P* < 0.001, *F*_4,22_ = 12.479), Tukey *post hoc* test, *n* = 4 to 5 per group) (Figure 2D).

When estimating the density of TH- and DAT-reactive fibers in the rat striatum, the two different doses of 6-OHDA resulted in highly significant unilateral decrease in the striatal fiber density at both 2 and 4 weeks post-lesion as compared to the intact rat striatum (one-way ANOVA (*P* < 0.001, *F*_4,22_ = 32.564 (TH) and *F*_4,22_ = 52.435 (DAT)) and Tukey *post hoc* test, *n* = 4 to 5 per group) (Figure 2B,C; representative pictures in Figure 1C,D). The extent of TH or DAT fiber density loss did not differ between the lesion groups (approximately 72% to 77% loss following 8 μg 6-OHDA and 86% to 90% following 2 × 10 μg 6-OHDA). No clear progression of the lesion could be seen in either TH-reactive cell counts or TH- or DAT-reactive fiber density measurements.

### Correlations

As expected, a high degree of positive correlation was found between the striatal DAT binding of [^123^I]β-CIT and measurements of TH- and DAT-reactive fiber densities in the rat striatum (Pearson correlation, *P* < 0.0001 at both time points) (Figure 3). Results from [^123^I]β-CIT SPECT/CT showed also high positive correlation to TH-reactive cell counts at both 2 and 4 weeks post-lesion (Figure 3). At 4 weeks post-lesion, the amphetamine-induced rotation asymmetry showed moderate negative correlation with the other measurements, while at 2 weeks post-lesion no correlations could be found between ipsilateral turning behavior and other data (Table 2).

## Discussion

The aim of our study was to estimate how well *in vivo* [^123^I]β-CIT SPECT/CT can discriminate the severity of dopaminergic dysfunction initiated by different intrastriatal 6-OHDA injections in rats. The unilateral partial 6-OHDA lesion model of PD has been widely used in pre-clinical studies of therapeutic interventions, but to our knowledge, the model has not yet been evaluated in both dose- and time-dependent manner with small-animal SPECT/CT using the DAT ligand [^123^I]β-CIT. Our results show that [^123^I]β-CIT SPECT/CT can reliably be used to distinguish between single- and two-site intrastriatal 6-OHDA injections and that the striatal binding of [^123^I]β-CIT correlates well with immunohistochemical findings.

Radioligands used for imaging in PD research focus on the dopaminergic system (for review, see [23]). Common tracers are [^18^F]l-3,4-dihydroxyphenylalanine (DOPA), that give an estimate of dopamine synthesis and radioligands with affinity for proteins important for the dopaminergic transmission (e.g., DAT, D_2_ receptor, vesicular monoamine transporter type 2). In a recent study, Kyono et al. [25] showed that PET detection of striatal uptake of [^18^F]FDOPA, after inhibition of aromatic l-amino acid decarboxylase and catechol-*O*-methyltransferase, is significantly correlated to striatal dopamine levels in the partially 6-OHDA-lesioned rat brain. They did four-site striatal 6-OHDA lesions of a total of 7, 14, or 28 μg 6-OHDA. The [^18^F]FDOPA uptake was significantly reduced in all three 6-OHDA groups as compared to vehicle-injected rats, but there was no significant difference in [^18^F]FDOPA uptake between the different 6-OHDA lesion groups using six animals per group. Uptake of DOPA (which includes transport, decarboxylation, and storage) can be affected by compensatory mechanisms in PD and PD models, leading to changes in the turnover [26,27]. In a study by Forsback et al. [28], the measured uptake of [^18^F]FDOPA showed less sensitivity and weaker correlation to nigral cell loss than the DAT tracer [^18^F]2-β-carbomethoxy-3β-(4-fluorophe-nyl)tropane ([^18^F]CFT) in an *ex vivo* study of 6-OHDA-lesioned rats. Compared to β-CIT, CFT is more selective for DAT since β-CIT also has affinity for the serotonin transporter (SERT) [29,30].

Most of the studies examining DAT binding *in vivo* have been conducted on rats that have received a unilateral injection of 6-OHDA to the MFB or SN [13-18,20-22]. In these studies, information about correlations between DAT binding and immunohistochemical findings are largely lacking, and immunohistochemistry has mainly been used as a tool to confirm the dopaminergic lesion after image acquisition. Unilateral injection of 8 μg 6-OHDA into the MFB led to an approximately 59% decrease in ipsilateral binding of [^123^I]β-CIT in the striatum, and the imaging data were highly correlated to TH-reactive cell counts in the SN [14]. When the same amount of 6-OHDA was injected into the SN, an approximately 29% decrease in striatal DAT binding of [^123^I]-*N*-ω-fluoropropyl-CIT ([^123^I]FP-CIT) was observed [13]. Injection of a larger dose of 6-OHDA (24 μg) lead to a 74% decrease in the [^123^I]FP-CIT binding [15].

In studies using PET cameras and tracers, decreases in striatal DAT binding ranging from 33% to 85% have been reported following injection of 8 μg 6-OHDA to the MFB of rats [17,20-22]. In addition, Inaji [20] and Pellegrino [21] and their co-workers showed that the decrease in binding was significantly correlated to drug-induced rotational behavior. In the SN 6-OHDA lesion model, ipsilateral DAT binding was decreased by 50% and 65% to 85% following injection of 4 μg [16] and 8 μg 6-OHDA [18], respectively. Moreover, Hume and co-workers [16] showed that the unilateral 6-OHDA injection did not affect the contralateral DAT binding despite the compensatory changes that are known to take place in the 6-OHDA model [5]. However, Van Camp et al*.*[15] reported a somewhat higher (although not significant) striatal activity of [^123^I]FP-CIT in the contralateral side of 6-OHDA-lesioned rats as compared to intact animals. When comparing the right (intact) side of 6-OHDA-treated and intact animals, we did not see any changes in striatal [^123^I]β-CIT binding, indicating that possible compensatory mechanisms did not affect the DAT binding activity in our assay.

Using the partial lesion model, with intrastriatal injection of 4 × 6 μg 6-OHDA, Cicchetti and co-workers [19] observed an approximately 65% decrease in striatal [^11^C]CFT uptake, while the DAT binding was not affected by a sham lesion. Sun et al. [31] reported a 24% decrease in [^11^C]CFT activity *ex vivo* in the ipsilateral striatum following intrastriatal injection of a total of 28 μg (4 × 7 μg) 6-OHDA. This is in line with our study in which intrastriatal injection of 2 × 10 μg resulted in an approximately 30% decrease in DAT density measured as the uptake of [^123^I]β-CIT. The loss of DAT (and TH)-immunoreactive fiber density in the striatum was much more pronounced showing a decrease of approximately 86% to 90% in the lesioned side as compared to the contralateral side. The difference in DAT density measured by the uptake of [^123^I]β-CIT and DAT-immunoreactive fiber density is probably due to methodological issues, such as the area analyzed (dorsal versus whole striatum).

Methodological issues may also explain why the line plots from the correlation analysis did not intercept with zero in the *xy*-axis. Another reason for this could be striatal binding of [^123^I]β-CIT (specific or non-specific) not related to DAT, e.g., [^123^I]β-CIT has also affinity to SERT [29,30]. In the rat striatum, DAT concentration is several folds higher than the concentration of SERT, and in normal healthy rat, striatal tissue binding of [^123^I]β-CIT can be considered to be mediated mainly by DAT [30]. Even though SERT binding activity is decreased in the rat 6-OHDA model [32], there may still be a change in the proportion of striatal DAT versus SERT binding of [^123^I]β-CIT. In a recent PET study, Sossi et al. [33] observed changes in the non-specific background signal from [^11^C]-methylphenidate (DAT tracer) in the striatum of unilaterally 6-OHDA-lesioned rats. When compared to the reference region, additional non-specific background signal was detected from the striatum that was almost fully denervated by 6-OHDA injection. This increase in the background signal in the lesioned striatum could interfere with the evaluation of the degree of denervation and may cause over-estimation of striatal DAT density and function.

No progression of the lesion could be seen during 4 weeks with any of the used measurement methods. This is in line with previous studies that have shown that degeneration of dopaminergic terminals happens mainly during the first week post-lesion and is complete within 3 weeks after intrastriatal injection of 6-OHDA [8,34]. In the partial 6-OHDA lesion model, the onset of cell death in the SNpc starts between 1 and 2 weeks post-lesion [8,35]. When nigral dopaminergic cells are labeled pre-lesioning (e.g., retrograde labeling with fluoro-gold), the cell loss following intrastriatal injection of 6-OHDA continues to progress for an extensive period of time (>4 weeks) [8]. However, the expression of TH is highly regulated, and there is an acute repression of TH phenotype following 6-OHDA injection resulting in maximum loss of nigral TH-immunoreactive neurons at already 2 weeks post-injection [8]. TH immunohistochemistry can therefore be considered to be a reliable marker for DA cell loss starting from approximately 4 weeks post-lesion.

A great advantage with *in vivo* [^123^I]β-CIT SPECT/CT to more conventional methods is the possibility to follow repeatedly the condition of the midbrain dopaminergic system in living animals. To our knowledge, the present study is the first to assess DAT binding in the partially 6-OHDA-lesioned rat, using small-animal SPECT/CT imaging. With our nanoSPECT/CT system, the test-retest variability was in a recent study of striatal [^123^I]β-CIT binding in mice defined to be 9% [36]. Thus, this methodology is suggested to be capable of accurate and repeatable measurement of DAT binding using [^123^I]-β-CIT. In PD research, continuous evaluation of the animals is usually done by monitoring motor function in different behavioral test settings. The most sensitive behavioral test used for unilaterally lesioned rats is the detection of amphetamine-induced rotational asymmetry that can react to a 40% to 50% decrease in striatal dopamine levels and 30% to 50% loss of nigral cells in the SNpc [6]. Criticism of the test concerns the irrelevance to motor symptoms seen in PD and the fact that the test requires administration of a drug (dopamine agonist) to induce the behavior (for review, see [37]). Even if it is a sensitive test, it has also been observed that the degree of rotation asymmetry does not necessarily correlate with the degree of striatal dopamine depletion and dopaminergic denervation [6,37]. This is also seen in our study in which, at 2 weeks post-lesion, rats lesioned with 8 μg 6-OHDA showed higher amount of drug-induced rotations than rats lesioned with 2 × 10 μg 6-OHDA, even though other analyses indicated that 2 × 10 μg 6-OHDA resulted in a significantly bigger lesion.

Compared to the amphetamine-induced rotational test, many other tests used to evaluate motor function in 6-OHDA-lesioned rats require more extensive dopaminergic denervation [6] and are therefore not useful for the detection of very limited loss of dopamine neurons. In our hands, an intrastriatal injection of 8 μg 6-OHDA resulted in a 30% reduction in TH-positive cells in the SNpc. This is approximately the same extent of cell loss that has been estimated to be found at the time of onset of motor symptoms in Parkinson's disease (discussed in [38]). As diagnostic procedures improve, it would be important to be able to study also lesions of smaller magnitude to evaluate the effect of new neuroprotective treatments aimed at this stage of the disease. Our work shows that *in vivo* [^123^I]β-CIT SPECT/CT could be useful for this purpose.

Neuronal compensation mechanisms [5,7,27] and spontaneous recovery of motor function despite no improvement or re-innervation of the dopaminergic pathway are other problems that can affect the outcome of some behavioral test (discussed in [37]). This may explain the inconsistency in the amount of ipsilateral amphetamine-induced rotations in our study showing a decrease in the extent of rotational asymmetry in the 8-μg lesion group between 2 and 4 weeks post-lesion. Obviously, the over-interpretation of results could be partly avoided by the use of *in vivo* [^123^I]β-CIT SPECT/CT as a complement to behavioral tests in animal PD models. Compensatory mechanisms should, of course, also be taken into account in SPECT/CT analyses of dopaminergic neurons since the results can be affected by changes in DAT expression, dopamine turnover, and axonal sprouting. Last but not least, behavioral tests tend to result in rather big inter-individual variations and require therefore often numerous animals before differences between treatment groups can reliably be detected. Our work shows that with groups of only four to five animals, [^123^I]β-CIT SPECT/CT can give statistically significant differences in striatal [^123^I]β-CIT uptake between two different partial lesions of the dopaminergic system. The only other measurement that could discriminate between the two different lesions was loss of nigral TH-reactive cells.

## Conclusions

We report here the use of [^123^I]β-CIT SPECT/CT in evaluating the unilateral partial 6-OHDA lesion model in rats. Results from [^123^I]β-CIT SPECT/CT showed a high correlation to immunohistochemical findings and could reliably be used to estimate the severity of the 6-OHDA-induced dopaminergic lesion, showing numerous advantages compared to conventional analysis methods.

## Abbreviations

CFT: 2-β-carbomethoxy-3β-(4-fluorophenyl)tropane; CT: Computed tomography; DAT: Dopamine transporter; [123I]β-CIT: 2β-carbomethoxy-3β-(4-[^123^I]iodophenyl)tropane; [123I]FP-CIT: *N*-ω-fluoropropyl-2β-carbomethoxy-3β-(4-[^123^I]iodophenyl)tropane; i.p: Intraperitoneal; l-DOPA: l-3,4-dihydroxyphenylalanine; MFB: Medial forebrain bundle; PD: Parkinson's disease; PET: Positron emission tomography; SERT: Serotonin transporter; SNpc: Substantia nigra pars compacta; SPECT: Single-photon emission computed tomography; TH: Tyrosine hydroxylase; 6-OHDA: 6-hydroxydopamine

## Competing interests

The authors declare that they have no competing interests.

## Authors' contributions

SB did the animal work, tissue processing, and morphometric analyses, participated in designing the study, and drafted the manuscript. MR, KB, and SB did the SPECT/CT experiments, and MR carried out the SPECT/CT data analysis and helped in drafting the manuscript. RKT and AR participated in the design of the study and revised the manuscript. AR participated in the immunohistochemical analysis. PTM and KB conceived the study, participated in its design and coordination, and revised the manuscript. All authors read and approved the final manuscript.

## References

[B1] Dauer W Przedborski S Parkinson's disease: mechanisms and models Neuron 2003 3 889 909 10.1016/S0896-6273(03)00568-3 12971891

[B2] Ungerstedt U 6-Hydroxy-dopamine induced degeneration of central monoamine neurons Eur J Pharmacol 1968 3 107 110 10.1016/0014-2999(68)90164-7 5718510

[B3] Ungerstedt U Arbuthnott GW Quantitative recording of rotational behavior in rats after 6-hydroxy-dopamine lesions of the nigrostriatal dopamine system Brain Res 1970 3 485 493 10.1016/0006-8993(70)90187-3 5494536

[B4] Schwarting RK Huston JP The unilateral 6-hydroxydopamine lesion model in behavioral brain research. Analysis of functional deficits, recovery and treatments Prog Neurobiol 1996 3 275 331 10.1016/S0301-0082(96)00040-8 8971983

[B5] Schwarting RK Huston JP Unilateral 6-hydroxydopamine lesions of meso-striatal dopamine neurons and their physiological sequelae Prog Neurobiol 1996 3 215 266 10.1016/S0301-0082(96)00015-9 8878304

[B6] Kirik D Rosenblad C Björklund A Characterization of behavioral and neurodegenerative changes following partial lesions of the nigrostriatal dopamine system induced by intrastriatal 6-hydroxydopamine in the rat Exp Neurol 1998 3 259 277 10.1006/exnr.1998.6848 9710526

[B7] Deumens R Blokland A Prickaerts J Modeling Parkinson's disease in rats: an evaluation of 6-OHDA lesions of the nigrostriatal pathway Exp Neurol 2002 3 303 317 10.1006/exnr.2002.7891 12061862

[B8] Sauer H Oertel WH Progressive degeneration of nigrostriatal dopamine neurons following intrastriatal terminal lesions with 6-hydroxydopamine: a combined retrograde tracing and immunocytochemical study in the rat Neuroscience 1994 3 401 415 10.1016/0306-4522(94)90605-X 7516500

[B9] Neumeyer JL Wang SY Milius RA Baldwin RM Zea-Ponce Y Hoffer PB Sybirska E al-Tikriti M Charney DS Malison RT [^123^I]-2 beta-carbomethoxy-3 beta-(4-iodophenyl)tropane: high-affinity SPECT radiotracer of monoamine reuptake sites in brain J Med Chem 1991 3 3144 3146 10.1021/jm00114a027 1920365

[B10] Innis RB Seibyl JP Scanley BE Laruelle M Abi-Dargham A Wallace E Baldwin RM Zea-Ponce Y Zoghbi S Wang S Single photon emission computed tomographic imaging demonstrates loss of striatal dopamine transporters in Parkinson disease Proc Natl Acad Sci U S A 1993 3 11965 11969 10.1073/pnas.90.24.11965 8265656 PMC48106

[B11] Cass WA Zahniser NR Flach KA Gerhardt GA Clearance of exogenous dopamine in rat dorsal striatum and nucleus accumbens: role of metabolism and effects of locally applied uptake inhibitors J Neurochem 1993 3 2269 2278 10.1111/j.1471-4159.1993.tb07469.x 8245977

[B12] Lorang D Amara SG Simerly RB Cell-type-specific expression of catecholamine transporters in the rat brain J Neurosci 1994 3 4903 4914 8046459 10.1523/JNEUROSCI.14-08-04903.1994 PMC6577178

[B13] Booij J de Bruin K Habraken JB Voorn P Imaging of dopamine transporters in rats using high-resolution pinhole single-photon emission tomography Eur J Nucl Med 2002 3 1221 1224 10.1007/s00259-002-0845-y 12418461

[B14] Scherfler C Donnemiller E Schocke M Dierkes K Decristoforo C Oberladstatter M Kolbitsch C Zschiegner F Riccabona G Poewe W Wenning G Evaluation of striatal dopamine transporter function in rats by in vivo beta-[^123^I]CIT pinhole SPECT NeuroImage 2002 3 128 141 10.1006/nimg.2002.1158 12482072

[B15] Van Camp N Vreys R Van Laere K Lauwers E Beque D Verhoye M Casteels C Verbruggen A Debyser Z Mortelmans L Sijbers J Nuyts J Baekelandt V Van der Linden A Morphologic and functional changes in the unilateral 6-hydroxydopamine lesion rat model for Parkinson's disease discerned with microSPECT and quantitative MRI MAGMA 2010 3 65 75 10.1007/s10334-010-0198-7 20169465

[B16] Hume SP Lammertsma AA Myers R Rajeswaran S Bloomfield PM Ashworth S Fricker RA Torres EM Watson I Jones T The potential of high-resolution positron emission tomography to monitor striatal dopaminergic function in rat models of disease J Neurosci Methods 1996 3 103 112 8872875

[B17] Chen YC Galpern WR Brownell AL Matthews RT Bogdanov M Isacson O Keltner JR Beal MF Rosen BR Jenkins BG Detection of dopaminergic neurotransmitter activity using pharmacologic MRI: correlation with PET, microdialysis, and behavioral data Magn Reson Med 1997 3 389 398 10.1002/mrm.1910380306 9339439

[B18] Nguyen TV Brownell AL Iris Chen YC Livni E Coyle JT Rosen BR Cavagna F Jenkins BG Detection of the effects of dopamine receptor supersensitivity using pharmacological MRI and correlations with PET Synapse 2000 3 57 65 10.1002/(SICI)1098-2396(200004)36:1<57::AID-SYN6>3.0.CO;2-K 10700026

[B19] Cicchetti F Brownell AL Williams K Chen YI Livni E Isacson O Neuroinflammation of the nigrostriatal pathway during progressive 6-OHDA dopamine degeneration in rats monitored by immunohistochemistry and PET imaging Eur J Neurosci 2002 3 991 998 10.1046/j.1460-9568.2002.01938.x 11918659

[B20] Inaji M Okauchi T Ando K Maeda J Nagai Y Yoshizaki T Okano H Nariai T Ohno K Obayashi S Higuchi M Suhara T Correlation between quantitative imaging and behavior in unilaterally 6-OHDA-lesioned rats Brain Res 2005 3 136 145 10.1016/j.brainres.2005.09.055 16298352

[B21] Pellegrino D Cicchetti F Wang X Zhu A Yu M Saint-Pierre M Brownell AL Modulation of dopaminergic and glutamatergic brain function: PET studies on parkinsonian rats J Nucl Med 2007 3 1147 1153 10.2967/jnumed.106.037796 17574972

[B22] Zhu A Wang X Yu M Wang JQ Brownell AL Evaluation of four pyridine analogs to characterize 6-OHDA-induced modulation of mGluR5 function in rat brain using microPET studies J Cereb Blood Flow Metab 2007 3 1623 1631 10.1038/sj.jcbfm.9600461 17299451

[B23] Nikolaus S Larisch R Vosberg H Beu M Hautzel H Wirrwar A Mueller HW Antke C In vivo imaging neurotransmitter function. The rat 6-hydroxydopamine model and its relevance for human Parkinson's disease Nuklearmedizin 2011 3 155 166 10.3413/Nukmed-0371-10-12 21409317

[B24] Paxinos G Watson C The Rat Brain in Stereotaxic Coordinates 1997 San Diego: Academic Press

[B25] Kyono K Takashima T Katayama Y Kawasaki T Zochi R Gouda M Kuwahara Y Takahashi K Wada Y Onoe H Watanabe Y Use of [^18^F]FDOPA-PET for *in vivo* evaluation of dopaminergic dysfunction in unilaterally 6-OHDA-lesioned rats EJNMMI Res 2011 3 25 10.1186/2191-219X-1-25 22214344 PMC3251329

[B26] Bernheimer H Birkmayer W Hornykiewicz O Jellinger K Seitelberger F Brain dopamine and the syndromes of Parkinson and Huntington. Clinical, morphological and neurochemical correlations J Neurol Sci 1973 3 415 455 10.1016/0022-510X(73)90175-5 4272516

[B27] Zigmond MJ Abercrombie ED Berger TW Grace AA Stricker EM Compensations after lesions of central dopaminergic neurons: some clinical and basic implications Trends Neurosci 1990 3 290 296 10.1016/0166-2236(90)90112-N 1695406

[B28] Forsback S Niemi R Marjamäki P Eskola O Bergman J Grönroos T Haaparanta M Haapalinna A Rinne J Solin O Uptake of 6-[^18^F]fluoro-L-dopa and [^18^F]CFT reflect nigral neuronal loss in a rat model of Parkinson's disease Synapse 2004 3 119 127 10.1002/syn.10293 14618679

[B29] Bergström KA Halldin C Hall H Lundkvist C Ginovart N Swahn CG Farde L In vitro and in vivo characterisation of nor-beta-CIT: a potential radioligand for visualisation of the serotonin transporter in the brain Eur J Nucl Med 1997 3 596 601 9169564 10.1007/BF00841395

[B30] Booij J Knol RJ Reneman L de Bruin K Janssen AG van Royen EA Iodine-123 labelled nor-beta-CIT binds to the serotonin transporter *in vivo* as assessed by biodistribution studies in rats Eur J Nucl Med 1998 3 1666 1669 10.1007/s002590050346 9871099

[B31] Sun W Sugiyama K Fang X Yamaguchi H Akamine S Magata Y Namba H Different striatal D2-like receptor function in an early stage after unilateral striatal lesion and medial forebrain bundle lesion in rats Brain Res 2010 3 227 235 20043892 10.1016/j.brainres.2009.12.048

[B32] Weng SJ Shiue CY Huang WS Cheng CY Huang SY Li IH Tao CC Chou TK Liao MH Chang YP Ma KH PET imaging of serotonin transporters with 4-[^18^F]-ADAM in a Parkinsonian rat model Cell Transplantin press 10.3727/096368912X658683 23127756

[B33] Sossi V Dinelle K Jivan S Fischer K Holden JE Doudet D In vivo dopamine transporter imaging in a unilateral 6-hydroxydopamine rat model of Parkinson disease using ^11^C-methylphenidate PET J Nucl Med 2012 3 813 822 10.2967/jnumed.111.101436 22492730

[B34] Kim SR Chen X Oo TF Kareva T Yarygina O Wang C During M Kholodilov N Burke RE Dopaminergic pathway reconstruction by Akt/Rheb-induced axon regeneration Ann Neurol 2011 3 110 120 10.1002/ana.22383 21437936 PMC3137673

[B35] Marti MJ Saura J Burke RE Jackson-Lewis V Jimenez A Bonastre M Tolosa E Striatal 6-hydroxydopamine induces apoptosis of nigral neurons in the adult rat Brain Res 2002 3 185 191 10.1016/S0006-8993(02)03694-6 12468044

[B36] Pitkonen M Hippeläinen E Raki M Andressoo J-O Urtti A Männistö PT Savolainen S Saarma M Bergström K Advanced brain dopamine transporter imaging in mice using small animal SPECT/CT EJNMMI Res 2012 3 55 10.1186/2191-219X-2-55 23021250 PMC3519614

[B37] Meredith GE Kang UJ Behavioral models of Parkinson's disease in rodents: a new look at an old problem Mov Disord 2006 3 1595 1606 10.1002/mds.21010 16830310

[B38] Cheng HC Ulane CM Burke RE Clinical progression in Parkinson disease and the neurobiology of axons Ann Neurol 2010 3 715 725 10.1002/ana.21995 20517933 PMC2918373

